# Malignant Lacrimal Sac Tumours—Review of the Literature and Report of Own Experience

**DOI:** 10.3390/medicina61030533

**Published:** 2025-03-18

**Authors:** Olga Karłowska-Bijak, Grażyna Stryjewska-Makuch, Marta Michalak-Kolarz, Magdalena Marków, Grażyna Lisowska

**Affiliations:** 1 Department of Otorhinolaryngology and Laryngological Oncology in Zabrze, Medical University of Silesia, 40-055 Katowice, Poland; magda.markow@sum.edu.pl (M.M.); glisowska@sum.edu.pl (G.L.); 2 Department of Laryngology and Laryngological Oncology, Leszek Giec Upper-Silesian Medical Centre of the Silesian Medical University, 40-635 Katowice, Poland; makuch_mg@wp.pl (G.S.-M.); michalak.m26@gmail.com (M.M.-K.)

**Keywords:** lacrimal drainage system pathology, lacrimal sac neoplasm, lacrimal sac tumour, lacrimal sac malignancy

## Abstract

*Background and Objectives*: Tumours of the lacrimal drainage system are rare and most located in the lacrimal sac. The authors of this study aimed to conduct a literature review to find out which malignant tumours most often occurred in the lacrimal sac and what symptoms patients reported in the early stages of the disease. *Materials and Methods*: The PubMed database was searched for papers published between 2019 and 2024. The inclusion criteria were presence of an abstract, malignant lacrimal sac tumours, papers written in English, studies on humans, and case reports. The exclusion criteria were lack of an abstract, pathologies other than malignant tumours, including benign tumours of the lacrimal drainage system, malignant tumours of a part of the drainage system other than the lacrimal sac, papers in languages other than English, studies not involving humans, and no case report. No gender criterion was used. *Results*: Based on the data available in the literature, 31 studies were included in the article, describing 34 cases of malignant lacrimal sac tumours. Moreover, a case of a 58-year-old patient diagnosed with non-Hodgkin lymphoma was presented. *Conclusions*: As a result of the literature analysis, it was impossible to find any symptoms reported by patients with lacrimal sac tumours that would clearly suggest their diagnosis.

## 1. Introduction

The lacrimal sac is a part of the lacrimal apparatus. This system consists of the secretory part (lacrimal gland, accessory glands) and the lacrimal ducts (superior and inferior lacrimal puncta, superior and inferior lacrimal canaliculi opening into the common lacrimal canaliculus, lacrimal sac and nasolacrimal duct) [1,2]. Communication between the orbit and the nasal cavity occurs through the nasolacrimal duct, opening into the inferior nasal passage. The lacrimal sac itself is in the lacrimal sac fossa on the medial wall of the orbit, from which it is separated by the orbital septum. The dimensions of the lacrimal sac are 5.6–11.1 mm in length and 4–5 mm in diameter [2,3]. Histopathologically, tear ducts are made of loose connective tissue, which forms the lamina propria of the mucosa and submucosa. The lamina propria is lined with bi- and multilayer columnar epithelium near the conjunctiva, and with multilayer ciliated columnar epithelium near the nasal cavity [4]. Tumours of the lacrimal drainage system are rare and most located in the lacrimal sac [5,6]. Most of them are malignant tumours (55%), most often of epithelial origin, whereas the other cases are non-epithelial tumours: lymphomas and melanomas [5,7,8]. The anatomical location of the lacrimal sac makes it very difficult to make a quick diagnosis of cancer. Secondary nasolacrimal duct obstruction occurs because of pressure from the growing tumour, which causes tearing. Tearing is one of the most common symptoms reported by patients affected by lacrimal duct pathology, which is why lacrimal sac tumours are often treated as chronic inflammation [5,6,9,10,11]. The aim of our study was to systematically review the literature to determine the most common ethiopathology of malignant lacrimal sac tumours, their clinical presentation and preferable management modalities, as well as to present the authors’ clinical experience regarding the malignant tumours of the lower lacrimal ducts.

## 2. Materials and Methods

The literature review was conducted according to the PRISMA (Preferred Reporting Items for Systematic Reviews and Meta-Analyses) 2020 guidelines [12]. Two authors (O.K.B. and M.M.K.) independently conducted a comprehensive literature review using the electronic database PubMed. The database was searched for papers published between June 2019 and August 2024. Comprehensive electronic search strategies included the following MeSH terms: (“lacrimal sac”) AND (“drainage” OR “system” OR “pathology” OR “neoplasm” OR “tumour” OR “malignancy”). The search used a time criterion, i.e., papers published in the last 5 years. When conducting a literature review, the following inclusion criteria were used: (1) presence of an abstract, (2) malignant tumour of the lacrimal sac, (3) written in English, (4) studies on humans, (5) case report. The exclusion criteria included the following: (1) lack of an abstract, (2) pathologies other than malignant tumours, including benign tumours of the lacrimal drainage system, (3) malignant tumour of a part of the drainage system other than the lacrimal sac, (4) papers in a language other than English, (5) studies not involving humans, (6) no case report. No gender criterion was used. The results obtained from the four terms mentioned above were exported to EndNote 20. Duplicate articles were automatically removed using the “find duplicates” function. Two reviewers (O.K.B. and M.M.K.) independently checked all the titles and abstracts. In the case of disagreement, the relevant articles were discussed between the authors until reaching consensus. Full texts were then assessed. All studies that did not meet the inclusion criteria were excluded.

## 3. Results

**Figure 1:**
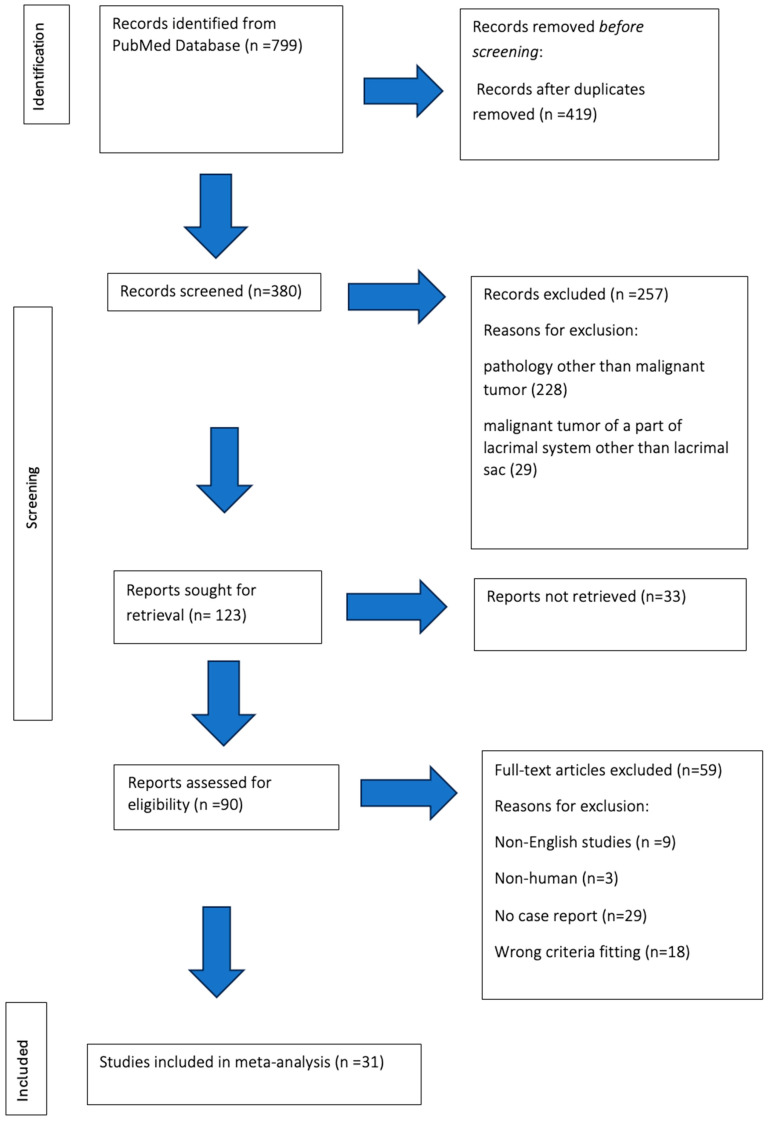
Flow diagram illustrating the literature selection process (adapted from the Preferred Reporting Items for Systematic Reviews and Meta-analysis (PRISMA).

The literature review yielded 799 articles. A total of 419 articles were removed during the primary verification process, which included identifying and excluding duplicates from the search. The remaining 380 articles were subjected to another verification and evaluation process. After reviewing the titles and abstracts, 257 publications were rejected due to the following reasons: 228 publications did not concern malignant lacrimal sac tumours, 29 publications concerned malignant tumours of a part of the drainage system other than the lacrimal sac. Case reports of malignant tumours of the lacrimal sac were searched for during the full-text review of 123 articles. A total of 33 publications were excluded due to the lack of access, nine publications were written in a language other than English, three publications concerned animals, 18 publications were not related to the topic of the article, and 29 did not include a case report. As a result, 31 publications were obtained for analysis (Figure 1).

All 31 articles were written in English and published between 2019 and 2024. They described 34 patients. The mean age of the patients was 56.5 years. All patients were diagnosed with malignant lacrimal sac tumours. The cancerous lesions were located on the right side in 21 patients, on the left side in 11 patients, and bilaterally in two patients. The most frequently reported symptom was excessive tearing in 24 cases, and in four patients it was the only symptom. Eight patients reported pain or tenderness in the lesion, whereas in seven described cases the area around the tumour mass was painless. Tissue swelling around the tumour occurred in 10 patients. A common symptom was inflammation of the lacrimal sac, sinuses, nasal cavity, or conjunctiva (eight cases). Eye symptoms such as proptosis, visual disturbances, decreased eyeball mobility, eyelid ptosis, and oculomotor nerve disorders were reported by six patients. Redness and change in skin colour affected men. In a 75-year-old patient, malignant melanoma manifested, in addition to tearing, as a lower eyelid lump and bleeding during the drainage of the lacrimal ducts. However, the only symptom reported by a 73-year-old woman with malignant melanoma was a sense of fullness of the medial angle of the eye.

Histopathological examination: Epithelial tumours (18 cases) were the most common histological type. Non-epithelial tumours concerned 15 cases, among which the most numerous were patients diagnosed with lymphoma 11. Four publications described cases of malignant lacrimal sac melanoma (Table 1).

Treatment: Patients diagnosed with epithelial tumours (15 cases) underwent surgical treatment, which in 12 cases was followed by chemotherapy and/or radiotherapy. In patients diagnosed with lacrimal sac lymphoma, surgical treatment was followed by chemotherapy and/or radiotherapy in eight cases, by chemotherapy alone in two cases, whereas in a 59-year-old woman with lymphoma, in addition to applying local and systemic treatment, a bone marrow transplant was performed. When malignant melanoma was diagnosed, surgical treatment was performed in two cases, and in one case it was also followed by radiotherapy and immunotherapy. The case report of a 60-year-old man with malignant melanoma did not include information on the treatment used (Table 2).

Post-treatment follow-up: among 14 patients who were under constant follow-up, no signs of tumour recurrence were found. In the case of a 58-year-old woman and a 63-year-old man, chronic symptoms persisted despite recovery. Similarly, a 59-year-old patient reported persistent excessive tearing. Disease stability was observed in four patients. A 55-year-old woman with squamous cell carcinoma experienced tumour recurrence after treatment. In the case of a 65-year-old woman with bilateral lymphoma, the tumour infiltration was reduced on both sides. Out of the patients described in the reviewed literature, three died, including one due to a cause other than a neoplastic disease. In the case of 10 articles, there was no information on health status after cancer treatment. The characteristics of the 31 included articles are summarised in Table S1.

The authors of this paper also presented their own experience with lacrimal sac tumours. In the Clinical Department of Otorhinolaryngology and Laryngological Oncology in Zabrze, in the period from 2017 to 2024, 126 operations were performed to unblock the lower tear ducts, consisting in exposing and wide opening of the lacrimal sac through the nasal cavity (dacryocystorhinostomy, DCR). One case of tumour was diagnosed.

Case report: In 2018, a 58-year-old patient was referred by an ophthalmologist to the Clinical Department of Otorhinolaryngology and Laryngological Oncology of the Specialist Hospital in Zabrze due to obstruction of the lower lacrimal ducts of the left eye. The patient reported persistent tearing, without pain or inflammation within the tear ducts. There was a history of adenoidectomy and tonsillectomy in childhood; in adulthood, the patient underwent endoscopic sinus surgery due to chronic inflammation without polyps. Moreover, in 2015, she underwent oncological treatment for papillary thyroid cancer. In July 2018, the patient underwent dacryocystorhinostomy (DCR) on the left side. There were no complications during the surgery. In 2020, the patient was admitted to the clinic again due to painful swelling of the frontal process of the maxilla and the cheek on the left side. The laryngological examination upon admission revealed significant pain when inserting the speculum into the left nasal passage and a bulging, swollen side wall of the nose. The mucous membrane was covered with purulent discharge and scabs. The nasal cavity on the right side was patent, with no signs of inflammation. There was no excessive tearing, visual disturbances, decreased eyeball mobility, or drooping of the eyelid of the left eye. No deviations from the normal condition were found in the ears, mouth, throat, and larynx. The lymph nodes in the neck were not enlarged. The patient was qualified for endoscopic surgery of the nasal cavities and paranasal sinuses. Based on the clinical picture and imaging studies, a hyperplastic process in the nasal cavity was suspected. On 30 January 2020, during the endoscopic surgery, very advanced necrotic and infiltrative lesions with bone destruction were found in the lacrimal sac and the frontal process of the maxilla. The following samples were taken for histopathological examination left inferior nasal concha, mucosa of the left nasal cavity, lateral wall of the nasal cavity, contents of the left maxilla, sections from the lacrimal sac, and nasolacrimal duct. There were no complications during the surgery. No nasal bleeding was observed immediately after the procedure. On the second postoperative day, the patient reported very severe pain in the left cheek area with a feeling of distension, as well as profuse sweating and weakness. The laryngological examination revealed tense, red, and swollen skin of the left cheek. No pathological discharge was revealed in the nasal cavities. Single, mobile, tender group IV neck lymph nodes were palpable on the left side. In laboratory tests, blood morphology and inflammation parameters did not show any significant deviations. Conservative treatment was initiated, and the patient’s condition improved. After receiving the results of the histopathological examination-non-Hodgkin lymphoma, the patient was referred for further treatment at an oncology centre.

## 4. Discussion

The aim of the literature review was to determine what symptoms reported by patients may suggest a lacrimal sac tumour to doctors of different medical specialties and what types of tumours are most common in this location. Pathologies of the lacrimal sac are at the intersection of interests of ophthalmologists, laryngologists, and maxillofacial surgeons. However, they are often neglected, hence the authors’ interest in this problem. The most frequently reported, although non-specific, symptom was epiphora [13,14,15,16,17,18,19,20,21,22,23,24,25,26,27,28,29,30,31,32,33,34,35,36,37,38,39,40,41,42,43]. It occurs in many diseases, both cancerous and non-cancerous [44]. Other, non-specific symptoms include pain, swelling, and redness of the skin in the lacrimal sac tumour [13,14,16,19,20,23,25,26,29,33,35,37,38,41,42]. Therefore, doctors should be particularly vigilant when patients report chronic ailments that do not subside after standard treatment. They may indicate a developing neoplastic disease. Cheng et al. described a patient with malignant lacrimal sac melanoma, in whom one of the symptoms was bleeding during the drainage of the lacrimal ducts [39]. This symptom is always a cause for concern. According to Kaushik et al., bloody tears are a rarely reported symptom, which is even more of a red flag for the specialist the patient sees [44]. Other symptoms that may raise oncological concerns are asymmetry in the medial canthal region or non-tender swelling of this region. Chu YC et al. emphasise the role of a detailed physical examination that can reveal asymmetry in the medial canthus region, which should prompt in-depth imaging diagnostics [45]. Despite a careful analysis of the literature, the authors were unable to find any symptoms reported by patients with lacrimal sac tumours that would clearly suggest this neoplastic disease in a timely manner. However, epiphora resistant to standard treatment, unexplained periorbital swelling, or recurrent dacryocystitis should raise suspicion for malignant lacrimal sac pathology. In such cases, Gleizal et al. suggest that the best diagnostic method is to perform a CT scan, which can detect a widening of lacrimal ducts occupied by a solid lesion [46].

In the analysed literature, patients with epithelial tumours, especially squamous cell carcinoma, were most frequently described. Among patients with non-epithelial tumours, lymphomas and malignant melanoma predominated (Table 1). In the authors’ own material, out of 126 lacrimal sac surgical procedures performed, there was one case of non-epithelial neoplasm, namely non-Hodgkin lymphoma. The described patient had painful swelling in the frontal process of the maxilla and cheek. The procedure described in the literature after diagnosing a malignant tumour is surgical treatment in most cases (27), followed by chemotherapy and/or radiotherapy (Table 2). In the case of the study presented by the authors, the patient underwent endoscopic surgical treatment and conservative treatment, and then, after obtaining a histopathological diagnosis, the patient was referred for further therapy at an oncology centre. Based on the review of the available literature, after diagnosing non-epithelial neoplasm, the most frequently used therapy was surgery with additional radio- and/or chemotherapy in nine cases (Table 2). Since no widely accepted treatment regimen for lacrimal sac malignancy exists, the choice of management modality should be primarily based on clinical presentation and disease stage.

Daniel L. Jones et al., when diagnosing NK/T-cell lymphoma in a 59-year-old patient, used allogeneic bone marrow transplantation in addition to surgery and radiochemotherapy [27]. David H Abramson et al. used, in addition to surgical treatment, androgen deprivation therapy in an 82-year-old patient diagnosed with lacrimal sac adenocarcinoma, obtaining a stable picture of the disease over 60 months of observation [22]. Diagnosis of cancer always involves a serious threat to the patient’s life and health. In the case of malignant lacrimal sac tumours, in 14 patients described in the literature, no tumour recurrence was observed after the therapy [13,17,18,19,20,24,26,28,30,31,35,37,38]. In two cases, the patients died due to malignancy [15,23].

In case of lacrimal sac squamous cell carcinomas (SCCs) diagnosis, the determination of active high-risk HPV infection might have a considerable impact on the preferred management modality [47]. Ogawa et al. described two cases with advanced HPV-related lacrimal sac carcinoma that were effectively treated with chemotherapy and chemoradiotherapy without the need for radical surgery [16]. The recent literature indicates that, in case of lymphoma suspicion, the use of immunohistochemistry (IHC) might be helpful in the determination of the final diagnosis, prognosis, and treatment plan. The most frequently determined markers in the non-Hodgkin lymphoma are CD20, CD3, CD5, CD10, BCL6, BCL2, Ki-67, MYC, and cyclin D1 [48]. Hans et al. developed an algorithm categorising DLBCL based on expression of CD10, BCL6, and MUM1 proteins, which was significantly associated with gene expression profiles and survival outcomes [49].

### Study Limitations

The present study has some limitations. Firstly, since our review comprises mainly non-randomised, retrospective studies with limited sample sizes, we advice caution in interpreting the results. Furthermore, the exclusion of non-English-language papers and searching solely one database could have restricted the already scarce literature on the topic. Regarding the presented case report, the detailed CT findings were unavailable during manuscript preparation due to the considerable time that had elapsed since the patient’s presentation (7 years). Furthermore, no results of biomarkers assessment could be retrieved. After patient referral to the oncological department, the patient was lost to follow-up at our department.

## 5. Conclusions

It can be concluded that early diagnosis and implementation of appropriate therapy give patients a good chance of complete recovery. However, based on the literature, it is difficult to clearly indicate typical symptoms, which could make doctors suspect a neoplastic disease. The rarity of this type of lesions in the drainage system, which often present symptoms similar to chronic lacrimal sac inflammation, may hinder a quick diagnosis. Therefore, specialists in the field of ophthalmology, laryngology, and maxillofacial surgery should be particularly vigilant, as they are the ones that patients with symptoms of the lacrimal drainage system are most often referred to.

The raw data supporting the conclusions of this article will be made available by the authors upon request.

## Figures and Tables

**Table 1:** Malignant tumours of the lacrimal sac.

Epithelial Tumours (n)	Non-Epithelial Tumours (n)	
Squamous cell carcinoma (12)	Lymphomas (11)	NKTCL (3)
Adenocarcinoma (3)		DLBCL (5)
Sebaceous carcinoma (1)		Low-grade B-cell lymphoma (1)
Adenoid cystic carcinoma (1)		MALT lymphoma (2)
Transitional cell carcinoma (1)	Malignant melanoma (4)	

**Table 2:** Treatment of malignant tumours of the lacrimal sac.

Epithelial tumours	Surgical treatment (2)
	Surgical treatment, chemotherapy and/or radiotherapy (12)
	Surgical treatment, androgen depravation therapy (1)
	Chemoradiotherapy (3)
	Chemotherapy, embolisation (1)
Non-epithelial tumours	Surgical treatment, chemotherapy and/or radiotherapy (9)
	Chemotherapy (2)
	Surgical treatment, chemoradiotherapy, bone marrow transplant (1)
	Surgical treatment (2)

## Supplementary Materials

The following supporting information can be downloaded at: https://www.mdpi.com/article/10.3390/medicina61030533/s1, Table S1: Characteristics of included articles.
