# Supplementary material for: Malignant Lacrimal Sac Tumours—Review of the Literature and Report of Own Experience
Source: Medicina (Kaunas). 2025 Mar 18;61(3):533. doi: 10.3390/medicina61030533 (PMC11943806; doi:10.3390/medicina61030533)
Supplement: Supplementary file 1 [file medicina-61-00533-s001.zip › medicina-3483164-supplementary.pdf]

Table S1. Characteristics of included articles.

Papers/Title	Lead author (year)	Number of cases	Sex	Age	Eye Right/ Left	Symphtoms	Histopatology	Treatment	Follow up
HER2-Positive Lacrimal Sac Squamous Cell Carcinoma in a 57-Year-Old Man	Nikolay Grachev et al.[13] (2024)	1	M	57	R	epiphora, painful mass in orbital region	squamous cell carcinoma (SCC)	endoscopic surgery resection of lacrimal sac tumor, part of the right orbital lamina and nasolacrimal duct, chemoradiotherapy, radiotherapy	no signs of recurrence follow-up period 18 months
**An Unusual Case of Extranodal Natural Killer/T-Cell Lymphoma, Nasal Type Masquerading as Dacryocystitis and Sinusitis**	Anthony N. Eze, BS et al.[14](2021)	1	M	38	L	epiphora, dacryocystitis, preseptal cellulitis, ethmoid and maxillary sinusitis, eriorbital pain facial–periorbital erythema and edema	extranodal nasal (NNKTL)	nasal endoscopy -biopsy and debridement of necrotizing sinonasal tissue, left maxillary antrostomy, left total ethmoidectomy, left frontal sinusotomy, repeat nasal endoscopy -left sinonasal debridement and biopsy of the left nare and left lateral nasal wall, chemoradiotherapy	no data
Natural killer T-cell lymphoma causing bilateral recurrent recalcitrant dacryocystitis.	Elzbieta Mechel et al.[15](2021)	1	F	35	L&R	recurrent, recalcitrant dacryocystitis Rhinosinusitis canaliculitis	extranodal nasal (NKTCL)	conservativ treatment, 2xFESS, DCR, incision and drainage left lacrimal sac, chemoradiotherapy	patient passed away to dead
A Novel Treatment Strategy for Advanced Lacrimal Sac Carcinomas Confirmed by p16 Immunostaining	Tetsuya Ogawa et al.[16] (2021)	2	M	71	R	swelling	squamous cell carcinoma (SCC)	chemoradiotherapy, radiotherapy, endonasal surgery	no data
			M	60	R	swelling with skin discoloration	squamous cell carcinoma (SCC)	chemoradiotherapy, radiotherapy	no data
Primary apocrine adenocarcinoma of the lacrimal sac: an extremely rare variant of	Md Shahid Alam et al. [17] (2020)	1	M	52	R	watering and painless gradual prominence	apocrine adenocarcinoma	surgery- sparing exenteration with bone removal in the area of possible bony invasion, radiotherapy	no signs of recurrence follow-up period 1 year

lacrimal sac neoplasm									
Lacrimal sac lymphoma: a case report	Somtaporn Ueathaweephol et al.[18] (2022)	1	F	71	R	painless mass below the right medial canthal area	diffuse large B-cell lymphoma (DLBCL)	chemotherapy	no signs of recurrence follow-up period 6 months
Chronic unilateral tearing as a sign of lacrimal sac squamous cell carcinoma	Aline Mota Freitas Matos et al. [19] (2021)	1	M	63	R	epiphora, acute inflammation of the medial canthal region, skin erythematous, pain	squamous cell carcinoma (SCC)	DCR, tumor excision with partial right maxillectomy and sinusotomy through lateral rhinotomy, radiotherapy	no signs of recurrence follow-up period 2 years
Nasolacrimal duct obstruction secondary to lacrimal sac involvement by sebaceous carcinoma	Albert Wu et al. [20] (2019)	1	F	57	R	epiphora, intermittent discomfort of the eye	sebaceous carcinoma (SC)	DCR, endoscopic approach- nasolacrimal duct access by excising the anterior head of the inferior turbinate, external incision- removed lacrimal drainage apparatus en bloc after freeing the canaliculi from the medial canthus, chemotherapy	no signs of recurrence follow-up period 3 months
Lacrimal sac primary squamous cell carcinoma with synchronous tonsillar primary squamous cell carcinoma	Zhiheng Lin et al. [21] (2019)	1	M	61	L	epiphora	squamous cell carcinoma (SCC)	external DCR, bilateral neck dissection, removal of the lids, nasal cartilage along with parts of the maxilla, nasal septum, middle turbinate, reconstruction anterolateral thigh free flap, radiotherapy	no data
Lacrimal sac adenocarcinoma managed with androgen deprivation	David H Abramson et al.[22] (2020)	1	M	82	R	epiphora, painless, palpable mass in the anterior orbit	adenocarcinoma	partial resection, androgen deprivation therapy	stable disease follow-up period 60 months
Epiphora and unrecognized paranasal sinuses pathology	Filippo Confalonieri et al.[23] (2020)	1	M	55	R	persistent unilateral epiphora, recurrent conjunctival infections, medial canthus and eyelids swelling.	squamous cell carcinoma (SCC)	external craniofacial resection by lateral rhinotomy and orbitectomy, reconstruction rectus abdominis free flap chemotherapy, radiotherapy	patient passed away to dead follow-up period 2 years
Poorly differentiated primary adenocarcinoma of the lacrimal sac and the nasolacrimal duct	Ashi Morawala et al.[24] (2019)	1	M	69	R	chronic dacryocystitis, mild abaxial proptosis, non-tender mass at and above the medial canthus	adenocarcinoma	en-block extended dacryocystectomy-combined external and endoscopic approach, radiotherapy	no signs of recurrence follow-up period 10 months

CT and MRI findings in relapsing primary malignant melanoma of the lacrimal sac: a case report and brief literature review	Ju-Wei Shao et al.[25] (2020)	1	F	50	L	mass in the lacrimal sac area, progressive swelling, epiphora, pain	malignant melanoma	DCR, total excision of tumor	no data
Primary mucosa-associated lymphoid tissue (MALT) lymphoma of the lacrimal drainage system in two pediatric patients.	Ahmet Yucel Ucgul et al.[26] (2019)	2	M	13	L	painless swelling, chronic epiphora	MALT lymphoma	external DCR, radiotherapy, chemotherapy	no signs of recurrence follow-up period 2 years
			F	12	L	slight painless stiffness, epiphora	MALT lymphoma	external DCR, radiotherapy	no signs of recurrence follow-up period 1 year
Recurrent natural killer/T-cell lymphoma of the lacrimal sac and nasolacrimal duct in a 59-year-old Caucasian woman.	Daniel L Jones et al.[27] (2019)	1	F	59	R	epiphora	NK/T-cell lymphoma	external DCR, radiotherapy, chemotherapy, allogeneic bone marrow transplant	patient reported persistent epiphora
Prelacrimal approach for nasolacrimal duct excision in the management of lacrimal system tumours.	David S Curragh et al.[28](2019)	1	F	58	R	epiphora	squamous cell carcinoma (SCC)	radiotherapy, surgery- combined external and endoscopic approach, reconstruction with a paramedian forehead flap	no signs of recurrence, persistent epiphora follow-up period 1 year
Primary non-Hodgkin diffuse large B-cell lymphoma of the lacrimal sac: a rare case of aggressive tumor and literature review	Maria Gabriella Malzone et al. [29] (2021)	1	F	77	L	epiphora, secretion upon awakening, heavy visual impairment, swelling of lacrimal sac region	non-Hodgkin lymphoma, diffuse large B-cell	conservative treatment, trans-cutaneous orbitotomy type surgery with an arched incision at the inferior internal orbital edge, chemotherapy, radiotherapy	no data
Successful management of adenoid cystic carcinoma of the lacrimal sac with	Shuyuan Zhang et al. [30] (2021)	1	M	30	L	epiphora, nasal congestion	adenoid cystic carcinoma	chemoradiotherapy	no signs of recurrence follow-up period 22 months

apatinib combined with concurrent chemoradiotherapy: a case report.									
Epithelial-Myoepithelial Carcinoma of the Lacrimal Sac and Literature Review of the Lacrimal System	Dhruv Sharma et al. [31] (2020)	1	M	72	R	epiphora, minimal proptosis	epithelial-myoepithelial carcinoma	maxillary antrostomy, partial ethmoidectomy, surgical excision of the neoplasm in a combined approach- right-sided combined transcaruncular, inferior transconjunctival orbitotomy and dacryocystectomy with subperiosteal dissection, endonasal resection, medial maxillectomy, anterior ethmoidectomy, inferior turbinectomy, and a partial middle turbinectomy	no signs of recurrence follow-up period 20 months
Melanoma of the lacrimal sac: An extremely rare location From a radiologist perspective	Anas Orgi et al. [32] (2024)	1	M	60	L	epiphora , chronic dacryocystitis, painless mass	malignant melanoma	no data	no data
Primary diffuse large B-cell lymphoma of the lacrimal sac with involvement of maxillary sinus: a case report and review of literature	Birkaran Sadhar et al. [33] (2024)	1	F	66	R	epiphora, infraorbital nerve hypesthesia, medial canthal swelling	diffuse large B-cell lymphoma (DLBCL)	chemotherapy, radiotherapy	follow up at 6-month- any growth of the tumor
Lacrimal sac Diffuse Large B-Cell Lymphoma Presenting as Sudden-Onset Binocular Diplopia	Antoine Jeri-Yabar et al. [34] (2024)	1	M	68	R	binocular diplopia, eye blurry vision, cranial third-nerve palsy	diffuse large B-cell lymphoma (DLBCL)	chemotherapy	no data
Lacrimal sac squamous cell carcinoma: From resection to prosthetic rehabilitation. A case report	Anna Poghosyan et al. [35] (2023)	1	M	30	R	slightly painful progressive mass in the area of the lacrimal sac, epiphora, acute dacryocystitis	squamous cell carcinoma (SCC)	conservative treatment, right-side wide en bloc resection with orbit exenteration including the ethmoid, lateral nasal bone, lacrimal sac and duct, and maxillary sinus upper wall	no signs of recurrence follow-up period 38 months
Transcatheter arterial infusion chemotherapy and embolization	Ming-Hui Sun et al. [36] (2022)	1	M	34	R	orbital mass with nasal obstruction	squamous cell carcinoma (SCC)	conservative treatment, arterial infusion chemotherapy, embolization	follow-up until April 2020 - lesion was stable

for primary lacrimal sac squamous cell carcinoma: A case report									
Value of ^{18}F-FDG PET/CT for Assessment of Advanced Lacrimal Sac Non-Keratinizing Squamous Cell Carcinoma Successfully Treated with Concurrent Chemoradiotherapy	Ching-Yu Liao, et al. [37] (2021)	1	F	71	R	medial orbital swellings, limited ocular mobility, drooping eyelids, blurred vision, persistent tearing	on-keratinizing squamous cell carcinoma (NKSCC)	chemoradiotherapy	no signs of recurrence follow-up period 23 months
Rare transitional cell carcinoma of the lacrimal sac.	Hayley V. Mille et al. [38] (2020)	1	M	63	R	dry eye and epiphora, tenderness of the upper eyelid, medial canthal mass	**transitional cell carcinoma**	DCR, surgical excision- complete right lacrimal sac resection with excision of entire medial canthal area, canalicular system, lacrimal sac, complete superior medial maxillectomy, radiotherapy, chemoradiotherapy, reconstructive surgery	no signs of recurrence, continuous tearing follow-up period 5 years
Radiotherapy for malignant melanoma of the lacrimal sac.	Tiffany W Cheng et al. [39] (2020)	2	F	73	L	fullness in the medial canthal region	malignant melanoma	subtotal exenteration of the orbit, superficial parotidectomy, selective neck dissection areas I and II, medial maxillectomy	disease-free until patient death 4 years later of a thoracic aneurysm unrelated to the malignant melanoma.
			M	75	R	lower eyelid bump, intermittent epiphora ,bloody drainage of the right eye.	malignant melanoma	DCR, oncologic resection, additional resection of right periorbital soft tissue, adjuvant proton beam radiotherapy, immunotherapy	no data
Navigation-assisted endoscopic surgery of lacrimal sac tumor	Cheng-Hsien Chang et al. [40] (2020)	1	F	55	R	epiphora	squamous cell carcinoma (SCC)	transnasal endoscopic surgery, chemotherapy, radiotherapy	recurrence of tumor 3 months after primary excision
An Unusual Presentation of Large B-cell Lymphoma	Joel Robinette et al. [41] (2019)	1	F	67	L	pruritic ovoid swelling, tearing, epiphora	large B-cell lymphoma	DCR, chemotherapy	stable since patient last office visit

From Seed to Spread: Lacrimal Sac Squamous Cell Carcinoma Blossoming Into Orbital Chaos	Chia Yaw Teoh et al. [42] (2024)	1	F	52	L	acute painful diminution of eye vision, intensive frontal headache	recurrent squamous cell carcinoma (SCC)	excision of tumor, completed radiotherapy modified enucleation with intraconal mass excised, palliative care	no data
Bilateral Primary Non-Hodgkin's Lymphoma of the Lacrimal Sac: A Case Report	Mohamed Noor Arjamilah et al. [43] (2022)	1	F	65	L&R	epiphora, recurrent acute dacryocystitis	low-grade B cell lymphoma	DCR, chemotherapy	reduction in the size of the bilateral nasolacrimal sac and duct soft tissue masses
